# Supplementary material for: The effect of intrapartum prolonged oxygen exposure on fetal metabolic status: secondary analysis from a randomized controlled trial
Source: Front Endocrinol (Lausanne). 2023 Jun 27;14:1204956. doi: 10.3389/fendo.2023.1204956 (PMC10335765; doi:10.3389/fendo.2023.1204956)
Supplement: Supplementary file 3 [file DataSheet_3.zip › Result-X101SC21092977-Z01-J001-B1-42/2.MetAnnotation/HMDB/meta_neg.HMDB.Anno.pdf]

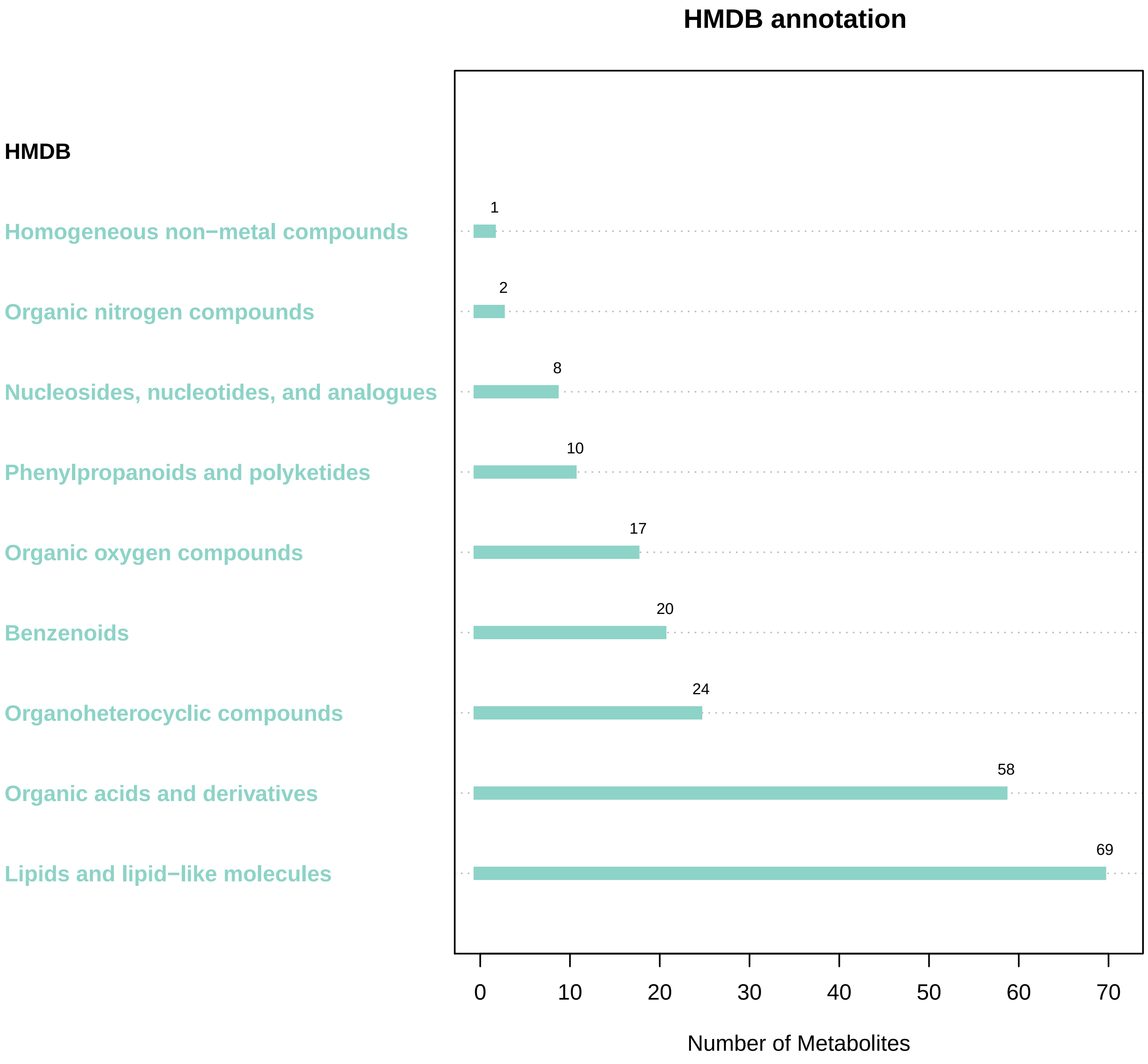
HMDB annotation
HMDB
Homogeneous non−metal compounds
Organic nitrogen compounds
Nucleosides, nucleotides, and analogues
Phenylpropanoids and polyketides
Organic oxygen compounds
Benzenoids
Organoheterocyclic compounds
Organic acids and derivatives
Lipids and lipid−like molecules
1
2
8
10
17
20
24
58
69
0
10
20
30
40
50
60
70
Number of Metabolites
